# Activation of cardiac AMPK-FGF21 feed-forward loop in acute myocardial infarction: Role of adrenergic overdrive and lipolysis byproducts

**DOI:** 10.1038/s41598-019-48356-1

**Published:** 2019-08-14

**Authors:** Hiroaki Sunaga, Norimichi Koitabashi, Tatsuya Iso, Hiroki Matsui, Masaru Obokata, Ryo Kawakami, Masami Murakami, Tomoyuki Yokoyama, Masahiko Kurabayashi

**Affiliations:** 1 Department of Cardiovascular Medicine, Gunma University Graduate School of Medicine, Maebashi, Gunma, Japan; 2 Department of Laboratory Sciences, Gunma University Graduate School of Health Sciences, Maebashi, Gunma, Japan; 3 Department of Clinical Laboratory Medicine, Gunma University Graduate School of Medicine, Maebashi, Gunma, Japan

**Keywords:** Mechanisms of disease, Myocardial infarction

## Abstract

Fibroblast growth factor 21 (FGF21) is a metabolic hormone having anti-oxidative and anti-hypertrophic effects. However, the regulation of FGF21 expression during acute myocardial infarction (AMI) remains unclear. We tested blood samples from 50 patients with AMI and 43 patients with stable angina pectoris (sAP) for FGF21, fatty acid binding protein 4 (FABP4), a protein secreted from adipocytes in response to adrenergic lipolytic signal, and total and individual fatty acids. Compared with sAP patients, AMI patients had higher serum FGF21 levels on admission, which were significantly correlated with peak FABP4 and saturated fatty acids (SFAs) but not with peak levels of cardiac troponin T. In mice, myocardial ischemia rapidly induced FGF21 production by the heart, which accompanied activation of AMP-activated protein kinase (AMPK)-dependent pathway. Like AICAR, an activator of AMPK, catecholamines (norepinephrine and isoproterenol) and SFAs (palmitate and stearate) significantly increased FGF21 production and release by cardiac myocytes via AMPK activation. Recombinant FGF21 induced its own expression as well as members of down-stream targets of AMPK involved in metabolic homeostasis and mitochondrial biogenesis in cardiac myocytes. These findings suggest that adrenergic overdrive and resultant adipose tissue lipolysis induce cardiac AMPK-FGF21 feed-forward loop that potentially provides cardioprotection against ischemic damage.

## Introduction

Myocardial ischemia induces a cellular process leading to not only adverse ventricular remodeling and serious arrhythmias but also potential healing of damaged myocardium at least partly through the induction of the heart-derived paracrine/autocrine factors that promote cytoprotection^1^. Activation of sympathetic nervous system (SNS) is a hallmark of the systemic response of the patients with acute myocardial infarction (AMI)^2–5^. Elevated levels of catecholamine and its lipolysis byproducts, free fatty acids (FFAs), are harmful to cardiac myocytes^6^ and may increase the risk of sudden death^7^. However, little is known about the effects of catecholamine and FFA on the production of cardioprotective hormone during AMI.

Fibroblast growth factor 21 (FGF21) is a pleiotropic peptide hormone that regulates energy metabolism in an autocrine, paracrine and endocrine manner, with main tissue of expression being the liver and adipose tissue^8^. Multiple studies reported that serum FGF21 levels in humans are elevated in obesity-related disease such as metabolic syndrome, type 2 diabetes, and nonalcoholic fatty liver disease^9,10^. In addition, FGF21 expression is induced upon endoplasmic reticulum (ER) stress and mitochondrial dysfunction in a variety of tissues including heart^11–13^ and skeletal muscle^14,15^. Notably, the cardiac production of FGF21 is up-regulated in murine model of isoproterenol-induced cardiac hypertrophy^16^, and is associated with an induction of anti-oxidant gene expression in cardiac hypertrophy and heart failure^13^. However, change in circulating FGF21 levels in AMI patients and its underlying mechanisms remain poor understood.

The overall aim of this study was to determine the regulatory mechanisms of circulating and cardiac FGF21 levels during early stage of AMI. We compared the time course of serum levels of FGF21, fatty acid binding protein 4 (FABP4, also known as adipocyte P2 (aP2)^17^) which is a byproduct of adipose tissue lipolysis stimulated by catecholamine^18,19^, FFA, and cardiac troponin T (cTnT) after onset of AMI, and measured 24 distinct species of fatty acids to determine the potential mechanisms of induction of FGF21. In addition, we tested if AMP-activated protein kinase (AMPK), which plays a major role in cellular metabolic homeostasis^20^, acts as an upstream regulator and as a downstream target of FGF21 in cardiac myocytes. This study provides new insight into the role of AMPK-FGF21 feed-forward loop in the cardioprotective response upon acute myocardial ischemia where excessive adrenergic activation and lipolysis take place.

## Results

### Demographic characteristics of the study subjects

A total of 93 patients were enrolled in this study, of whom 43 were diagnosed as stable angina pectoris (AP), and 50 were as AMI. Demographic characteristics and clinical history of stable AP and AMI groups were summarized in Table 1. Age, gender, body mass index (BMI), systolic blood pressure, diastolic blood pressure, heart rate were similar between the two groups. The prevalence of hypertension, diabetes, dyslipidemia, smoking and family history of coronary artery disease was also comparable between the groups. Prior myocardial infarction was more prevalent in stable AP group. The AMI group was more likely to have higher serum LDL-cholesterol, alanine transaminase (ALT) and glucose levels compared to the stable AP group. The users of antiplatelet agents and statins were significantly more in the stable AP group than the AMI group.

**Table 1:** Comparison of the patients at admission between stable AP and AMI patients.

	Total (n = 93)	Stable AP (n = 43)	AMI (n = 50)	p
Age - years	70.5 ± 9.7	70.1 ± 8.3	70.4 ± 10.8	0.89
Male sex - no. (%)	74 (79.6)	32 (74.4)	42 (84.0)	0.25
BMI - kg/m^2^	23.3 ± 3.4	23.2 ± 3.9	23.3 ± 2.8	0.88
Systolic blood pressure – mmHg	133.9 ± 27.7	130.0 ± 23.5	137.3 ± 30.4	0.20
Diastolic blood pressure – mmHg	75.6 ± 18.0	72.3 ± 12.5	78.4 ± 21.3	0.09
Heart rate – bpm	75.4 ± 13.6	72.7 ± 11.7	77.6 ± 14.6	0.08
**Coronary Risk Factors**				
Hypertension - no. (%)	63 (67.7)	30 (70.0)	33 (66.0)	0.70
Diabetes mellitus - no. (%)	35 (37.6)	19 (44.1)	16 (32.0)	0.23
Dyslipidemia - no. (%)	60 (64.5)	31 (72.1)	29 (58.0)	0.16
Previous myocardial infarction - no. (%)	52 (55.9)	37 (86.0)	15 (30.0)	<0.001
Chronic kidney disease - no. (%)	17 (18.3)	3 (7.0)	14 (28.0)	0.009
Smoking - no. (%)	62 (66.7)	29 (67.4)	33 (66.0)	0.88
Family history - no. (%)	35 (37.6)	15 (34.9)	20 (40.0)	0.61
**Laboratory data**				
LDL-cholesterol - mg/dL	110.4 ± 34.7	101.2 ± 32.3	118.2 ± 34.8	0.02
HDL-cholesterol - mg/dl	46.0 ± 13.6	47.5 ± 12.3	44.7 ± 14.6	0.32
Triglycerides - mg/dl	126.5 ± 119.1	122.4 ± 72.4	130 ± 147.8	0.75
Non-HDL-C - mg/dL	135.7 ± 42.8	125.8 ± 40.2	144.2 ± 43.2	0.02
ALT - IU/l	30.3 ± 29.9	20.9 ± 12.3	37.8 ± 36.8	0.004
ALP - IU/l	253.7 ± 141.8	269.8 ± 196.0	241.6 ± 77.3	0.42
γGTP - IU/l	50.6 ± 56.6	47.1 ± 59.9	53.4 ± 53.7	0.62
eGFR, mL/min/1.73 m^2^	71.2 ± 22.7	68.9 ± 20.9	73.2 ± 23.9	0.36
Glucose - mg/dl	163.4 ± 73.9	139.1 ± 68.2	184.8 ± 72.0	0.003
Hemoglobin - g/dl	13.6 ± 2.0	13.2 ± 1.7	13.9 ± 2.2	0.09
FGF21 – pg/mL	732.8 ± 129.8	238.9 ± 35.6	1157.5 ± 225.0	<0.001
FABP4 - ng/mL	29.1 ± 2.9	18.6 ± 2.6	38.1 ± 4.7	<0.001
FFA - μEq/L	2219.5 ± 181.5	1415.6 ± 150.6	2910.9 ± 280.2	<0.001
**Medications**				
Antiplatelets - no. (%)	68 (73.1)	43 (100)	25 (50.0)	<0.001
Beta-blockers - no. (%)	31 (33.3)	18 (41.9)	13 (26.0)	0.11
Calcium channel blockers - no. (%)	51 (54.8)	18 (41.9)	33 (66.0)	0.02
ACEIs/ARBs - no. (%)	40 (43.0)	21 (48.8)	19 (38.0)	0.29
Statins - no. (%)	64 (68.8)	41 (95.3)	23 (46.0)	<0.001

Values represent the mean ± SD, or n (%).

BMI indicates body mass index; LDL, low-density lipoprotein; HDL, high-density lipoprotein; ALT, alanine transaminase; ALP, alkaline phosphatase; γGTP, gamma-glutamyl transferase; eGFR, estimated glomerular filtration rate; CK, creatinine kinase; FGF21, fibroblast growth factor; FABP4, fatty acid binding protein 4; FFA, free fatty acid; cTnT, cardiac troponin T, ACEIs/ARBs, angiotensin-converting enzyme inhibitors/angiotensin-receptor blockers.

### Time course of serum FGF21, FABP4, FFA and cTnT levels during initial stage of AMI

**Figure 1:**
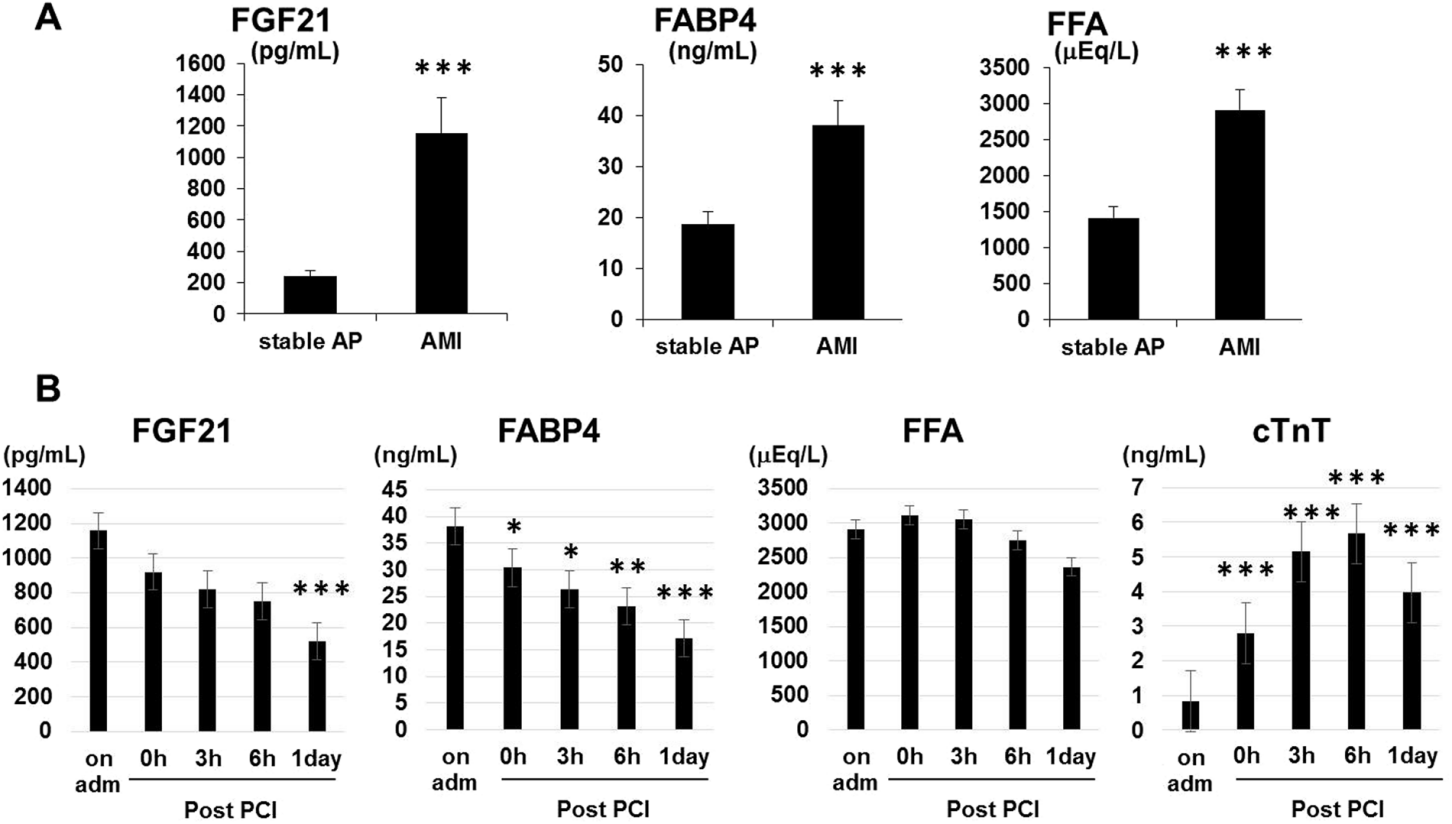
Serum concentrations of FGF21, FABP4 and FFA in patients with stable AP and AMI. (**A**) Serum concentrations of FGF21, FABP4 and FFA in the stable AP patients (n = 43) and AMI patients (n = 50) on admission. Results are represented as the means ± SEM. ***p < 0.001, as measured by Mann-Whitney *U*-test, or student *t*-test. (**B**) Time course of serum FGF21, FABP4, FFA and cTnT and FFA in patients with AMI Data are presented as the means ± SEM. *p < 0.05, **p < 0.01, ***p < 0.001 compared with samples on admission (adm). PCI: percutaneous coronary intervention.

Circulating FGF21 levels on admission were significantly higher in the AMI group compared to the stable AP group (1157.5 ± 225.0 pg/ml versus 238.9 ± 35.6 pg/ml, p < 0.001) (Fig. 1A). The concentrations of FABP4 (38.1 ± 4.4 ng/mL versus 18.6 ± 2.6 ng/mL, p < 0.001) and FFA (2910.9 ± 280.2 μEq/L versus 1415.6 ± 150.6 μEq/L, p < 0.001) were significantly higher in the AMI group compared to the stable AP group.

Figure 1B shows the time course of serum concentrations of FGF21, FABP4, FFA and cTnT during the first 24 h after admission in the AMI group. Both FGF21 and FABP4 concentrations peaked on admission, and they declined over time. In subgroup of AMI patients (n = 35) whose serial measurements of FGF21, FABP4, FFA and cTnT from hospital admission to 3 days after admission were avairable, we observed that FGF21 levels but not FABP4 and FFA levels at 3 days were still significantly higher than controls (patients with stable AP) (p < 0.05) (data not shown). In contrast, cTnT concentrations were increased after primary percutaneous coronary intervention (PCI), and declined afterwards (p < 0.001).

### Correlation between serum FGF21 and clinical and laboratory parameters

Results of the association analyses between peak FGF21 and clinical variables and laboratory parameters in the AMI group are presented in Table 2. Notably, univariate analyses revealed that peak FGF21 was significantly correlated with hemoglobin, TG, ALT, alkaline phosphatase (ALP), gamma-glutamyl transferase (γGTP) and peak FABP4 but not with peak FFA and peak cTnT. In multivariable regression analysis, peak FABP4 and TG were significantly correlated with peak FGF21 levels (β = 0.442, p = 0.005; β = 0.0007, p = 0.003, respectively) (Table 2).

**Table 2:** Univariate and multivariate regression analysis for FGF21.

	Univariate analysis		Multivariate analysis	
	r	p	β	p
Age	0.031	0.828		
BMI	0.167	0.074		
Systolic blood pressure	0.173	0.228		
Diastolic blood pressure	−0.073	0.616		
Hemoglobin	−0.292	0.010	−0.002	0.183
LDL-Cholesterol	−0.253	0.077		
HDL-Cholesterol	−0.024	0.870		
Triglyceride	0.344	0.014	0.0007	0.003
ALT	0.293	0.04	0.0007	0.575
ALP	0.406	0.003	0.0006	0.316
γGTP	0.459	<0.001	0.002	0.083
eGFR	−0.184	0.200		
Glucose	0.211	0.141		
CK	0.117	0.419		
Peak FABP4*	0.425	0.002	0.442	0.005
Peak FFA*	0.035	0.808		
Peak cTnT*	0.021	0.887		

BMI indicates body mass index; LDL, low-density lipoprotein; HDL, high-density lipoprotein; ALT, alanine transaminase; ALP, alkaline phosphatase; γGTP, gamma-glutamyl transferase; eGFR, estimated glomerular filtration rate; CK, creatinine kinase; FABP4, fatty acid binding protein 4; FFA, free fatty acid; cTnT, cardiac troponin T.

*Values are log-transformed before analysis.

### Comparison of the serum fatty acid profile between the stable AP and the AMI groups

Quantities of individual fatty acids molecular species in blood collected on admission (distinct 24 species listed in Table 3) were determined by gas chromatography and were expressed as a percentage of total fatty acids (Supplementary Table S1). The AMI group has higher levels of two major saturated fatty acids (palmitic acid (PA), stearic acid (SA)) and lower levels of unsaturated fatty acid oleic acid (OA), γ-linolenic acid. In addition, the AMI group has higher levels of arachidonic acid (AA, C20:4n-6) and lower levels of eicosapentaenoic acid (EPA, C20:5n-3) and docosapentaenoic acid (DHA, C22:5n-3), and thus, EPA/AA ratio, and DHA/AA ration, and EPA + DHA/AA ratio were significantly lower in the AMI group compared with the stable AP group.

**Table 3:** Univariate regression analysis for FGF21.

	Univariate analysis	
	r	p
C12:0	−0.036	0.778
C14:0	−0.119	0.35
C14:1n-5	−0.224	0.075
C16:0 (palmitic acid)	0.478	<0.001
C16:1n-7	−0.063	0.621
C18:0 (stearic acid)	0.267	0.033
C18:1n-9 (oleic acid)	−0.187	0.138
C18:2n-6 (linoleic acid)	−0.087	0.495
C18:3n-6 (γ-linolenic acid)	−0.369	0.003
C18:3n-3	−0.176	0.165
C20:0	0.129	0.309
C20:1n-9	0.142	0.262
C20:2n-6	0.294	0.018
C20:3n-9	−0.113	0.374
C20:3n-6	−0.134	0.292
C20:4n-6 (AA, arachidonic acid)	0.121	0.341
C20:5n-3 (EPA, eicosapentaenoic acid)	−0.222	0.079
C22:0	0.029	0.823
C22:1n-9	0.078	0.541
C22:4n-6	−0.36	0.003
C22:5n-3	−0.151	0.235
C24:0	0.036	0.777
C22:6n-3 (DHA, docosahexaenoic acid)	−0.269	0.032
C24:1n-9	0.12	0.347
EPA/AA	−0.226	0.072
DHA/AA	−0.279	0.026
(EPA ± DHA)/AA	−0.273	0.029

### Correlation between FGF21 and individual fatty acid concentrations

We examined the relationship between FGF21 and individual fatty acid concentrations in blood drawn on admission. As shown in Table 3, univariate analysis of 64 patients (30 patients in stable AP group, 34 patients in the AMI group) revealed that PA (C16:0), SA (C18:0) and C20:2n-6 levels were positively associated with FGF21 (r = 0.478, p < 0.001; r = 0.267, p = 0.033, r = 0.294, p = 0.018, respectively) while γ-linolenic acid (C18:3n-6), C22:4n-6, C22:6n-3 were negatively associated with FGF21 (r = −0.369, p = 0.003; r = −0.3604, p = 0.003, and r = −0.269, p = 0.032, respectively).

### Increase in serum and myocardial levels of FGF21 in mice model of AMI

**Figure 2:**
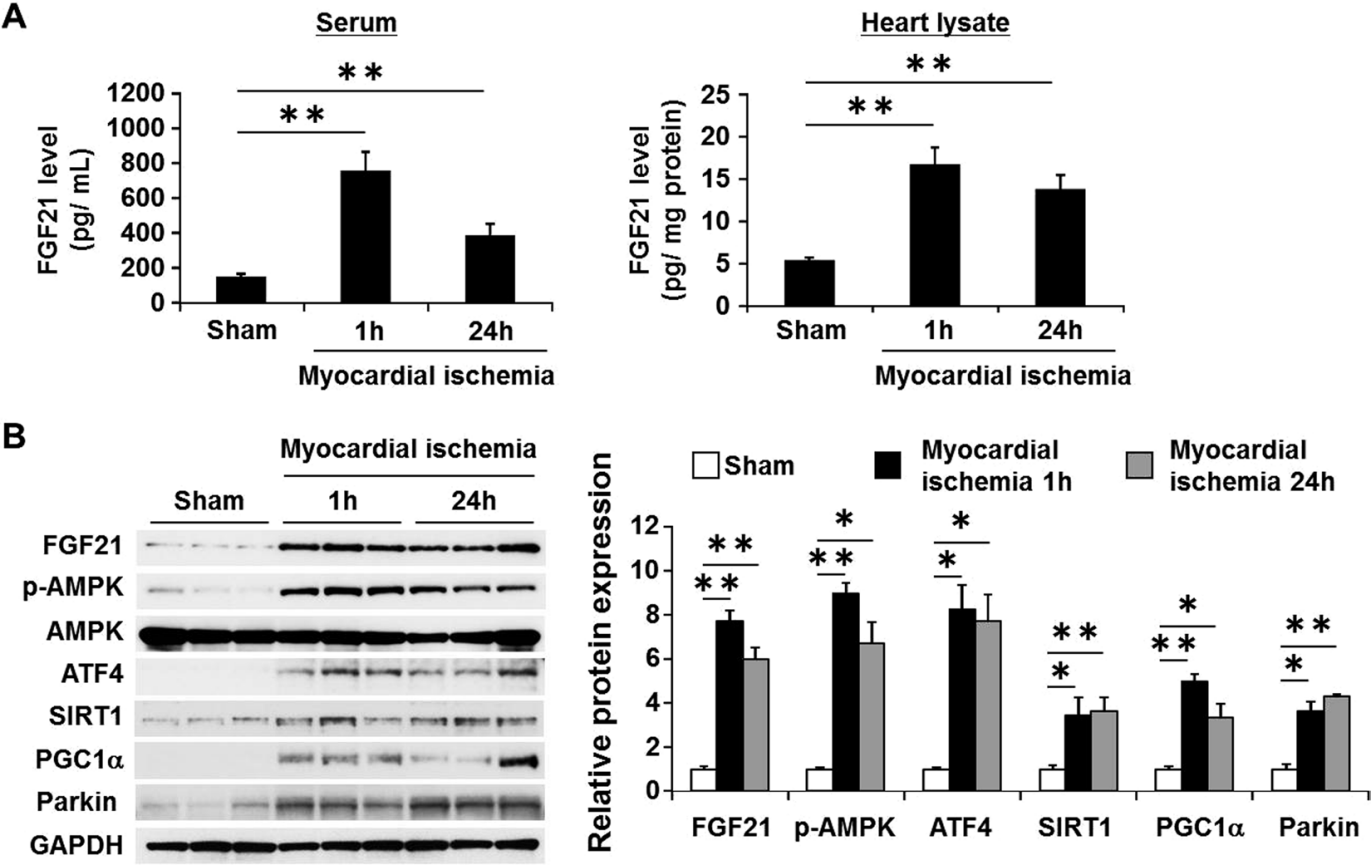
Serum FGF21 and cardiac FGF21 expression in myocardial ischemia in mice. (**A**) Results of ELISA assay of serum and heart lysate at 1 h (n = 6) and 24 h (n = 6) after myocardial ischemia or sham-operated mice (n = 6). (**B**) Representative western blots are shown on the left. Protein levels were normalized by GAPDH, and phosphorylation of proteins were normalized by total proteins in the same samples. Full-length blots are presented in Supplementary Fig. S1. Data are presented as mean ± SEM from 2 different sets of experiments on the right. Expression level of sham is expressed as 1.0. *p < 0.05, **p < 0.01, as measured by Tukey-Kramer test (**A**) or Steel-Dwass test (**B**).

To explore the mechanisms by which circulating FGF21 was increased during early hours of AMI, we set out the experiments using mice model of myocardial ischemia (Fig. 2A). Consistent with the findings in AMI patients, serum FGF21 concentrations were rapidly and robustly elevated within 1 h after coronary occlusion (4.6-fold, p < 0.01), and remained higher than control at 24 h and 1 week after coronary occlusion (data not shown). Notably, FGF21 levels in heart lysates were increased by 3.2-fold at 1 h (p < 0.01; n = 5), by 2.8-fold at 24 h (p < 0.01; n = 5) and by 1.8-fold at 1 week (p < 0.05; n = 3). Western blot analysis of heart tissue revealed that FGF21 protein levels markedly increased at 1 h (7.8-fold, p < 0.01) and remained elevated at 24 h and 1 week after coronary occlusion. These results indicate that production and release of FGF21 by the heart is robustly induced upon onset myocardial ischemia and persist for a good while.

In parallel with an induction of FGF21, Thr^172^ phosphorylation of AMPKα1 subunit, which is essential for AMPK activity, was robustly increased as determined by Western blot analysis using anti–phospho-AMPK antibody (8.1-fold, p < 0.01 at 1 h; 6.0-fold, p < 0.01 at 24 h) (Fig. 2B, Supplementary Fig. S1). In addition, ATF4, zinc-finger type of transcription factor that stimulates the expression of the genes for endoplasmic reticulum (ER) stress and anti-oxidative stress responses^21,22^ and Sirtuin 1 (SIRT1), a member of the sirtuin family of class III histone deacetylase that protects the heart from oxidative stress^23^ accompanied FGF21 induction and AMPK phosphorylation in ischemic myocardium. Notably, Parkin, the E3 ubiquitin ligase that plays a crucial role in the ischemic response during the early phase of myocardial infarction by enhancing degradation of damaged mitochondria via a specific form of autophagy termed mitophagy^24–26^, was markedly induced as early as 1 h after myocardial ischemia, when an induction of FGF21 was clearly observed.

### Induction of FGF21 expression by AICAR in cardiac myocytes

**Figure 3:**
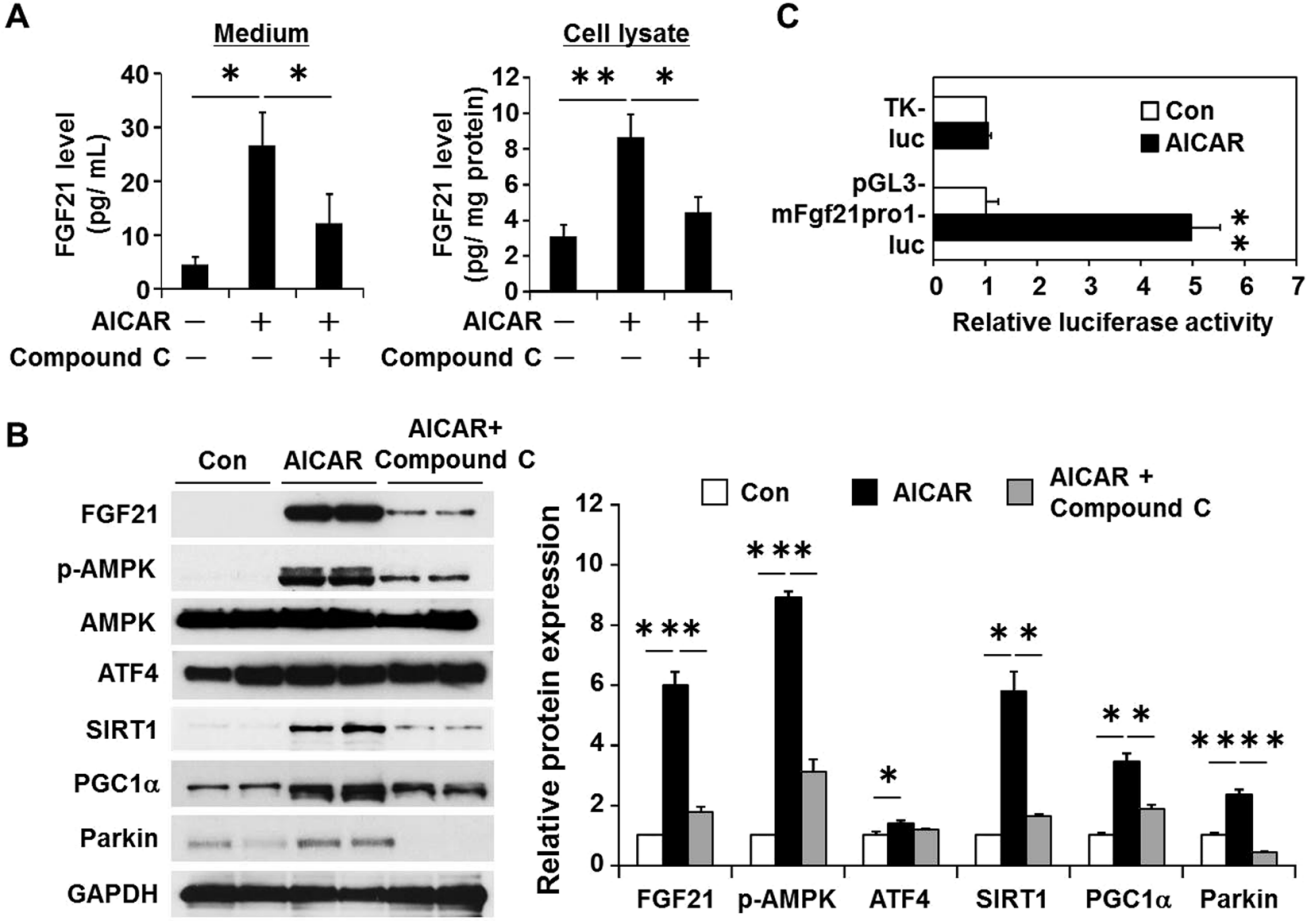
Effects of 5-aminoimidazole-4-carboxyamide ribofuranoside (AICAR) on FGF21 production in cultured neonatal rat cardiac myocytes. (**A**) FGF21 protein levels in cultured medium and cell lysates were measured by ELISA after stimulation of cultured neonatal rat cardiac myocytes with AICAR (1 mM) in the presence or absence of compound C (10 μM). (**B**) Representative Western blots are shown on the left. Full-length blots are presented in Supplementary Fig. S2. (**C**) Cultured neonatal rat cardiac myocytes were transfected with the luciferase reporter plasmid pGL3-mFgf21pro1-luc, which contains 1.5 kb of murine FGF21 promoter region in front of the luciferase gene or control plasmid (thymidine kinase-luc (TK-luciferase)). Cells were then treated with either vehicle or 1 mM AICAR for 24 h before harvest. Data are presented as mean ± SEM from 3 different sets of experiments. Expression level of control is expressed as 1.0. *p < 0.05, **p < 0.01, as measured by Tukey-Kramer test **(A**), Steel-Dwass test (**B**) or unpaired Student’s t-test (**C**).

To ascertain whether FGF21 expression is mediated by AMPK activation, we examined the effects of 5-aminoimidazole-4-carboxamide-1-β-D-ribofuranoside (AICAR), an activator of AMPK, on FGF21 expression in the cultured cardiac myocytes. AICAR (1 mM) results in a substantial increase in FGF21 released into cultured medium and FGF21 expressed in cell lysates as determined by ELISA (Fig. 3A) and by Western blot analysis (Fig. 3B, Supplementary Fig. S2). As expected, AICAR-induced FGF21 release and expression were profoundly attenuated in the presence of 10 μM of compound C, a potent selective AMPK inhibitor that is competitive with ATP^27^. A set of the genes, including SIRT1, PGC1α, and Parkin, that functions to protect the heart from ischemic insult, was induced by AICAR via AMPK activation.

To determine whether FGF21 expression is induced at the transcriptional level, pGL3-mFgf21 pro1-luc, containing 1.5 kb of murine FGF21 promoter sequence in front of the luciferase gene, was introduced into cardiac myocytes and those cells were stimulated with AICAR. Result showed that AICAR induced luciferase activity by ~5.0-fold, while no effects was observed in control vector (TK-luc), suggesting that AICAR stimulates the transcription of the FGF21 gene (Fig. 3C).

### Induction of FGF21 expression by catecholamines and SFAs via AMPK activation in cardiac myocytes

We next explored the molecular mechanisms of the induction of FGF21 expression using cultured neonatal rat cardiac myocytes. Our clinical data show that serum FGF21 concentrations in AMI patients are positively correlated with serum FABP4 and palmitic acid. Our previous study demonstrated that FABP4 is released from adipocytes by adrenergic stimulation, and serum FABP4 levels are correlated with those of serum norepinephrine during exercise. It is well documented that myocardial ischemia evokes systemic and cardiac sympathetic nervous system (SNS) activation and catecholamine release into circulation and into the acutely ischemic myocardium^2,28^. In addition, circulating FFA concentrations rise rapidly through the catecholamine-induced tissue lipolysis of stored glyceride^29^. Accordingly, we tested the role of catecholamine and free fatty acids in eliciting intracellular signaling pathway that leads to FGF21 expression in cardiac myocytes.

**Figure 4:**
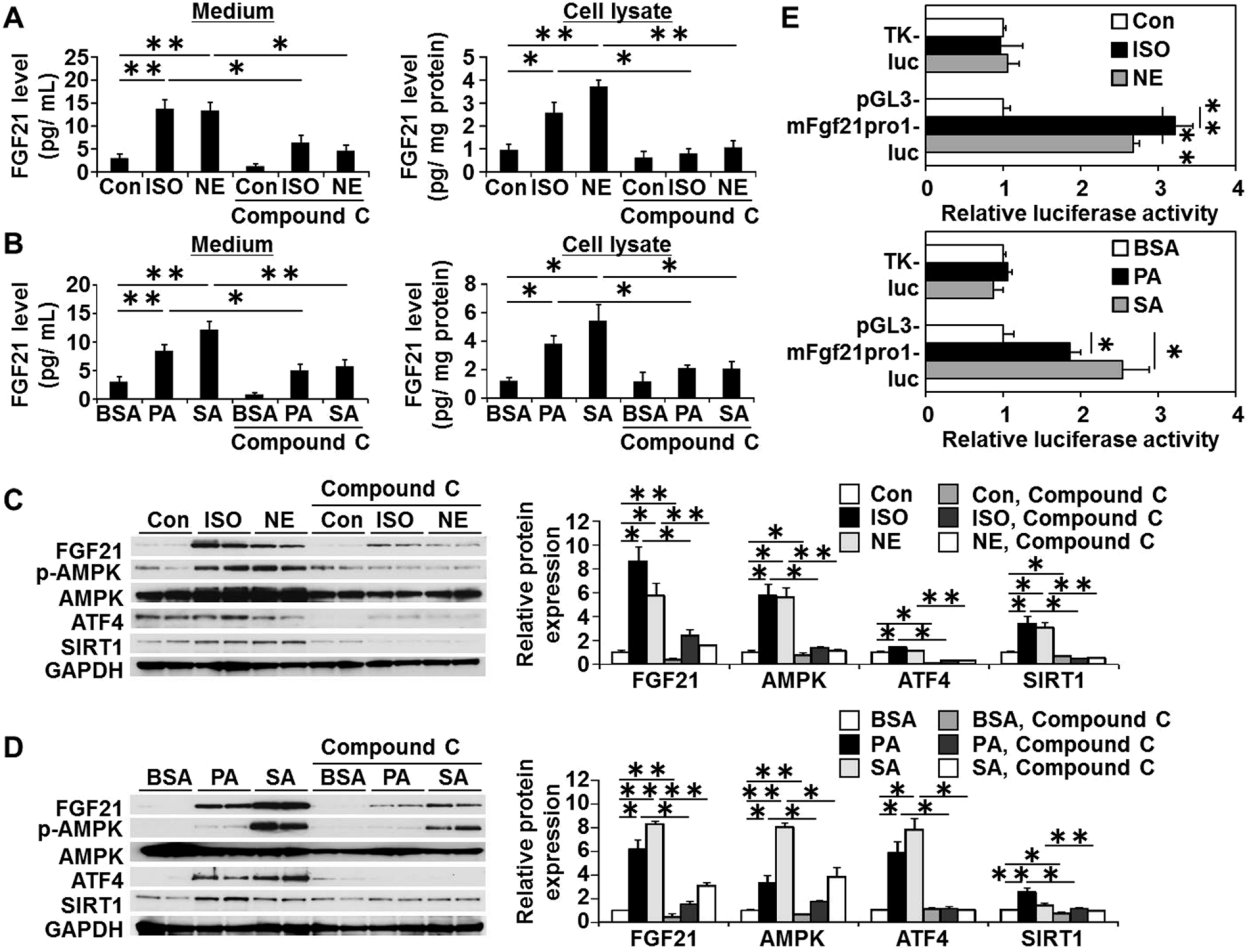
Induction of FGF21 production by catecholamines or SFAs in cultured neonatal rat cardiac myocytes. (**A–D**) Cultured neonatal rat cardiac myocytes were stimulated with a pan-β-adrenergic receptor agonist isoproterenol (ISO) (10 μM), norepinephrine (NE) (10 μM) or fatty acid-free bovine serum albumin (BSA)-conjugated palmitic acid (PA), stearic acid (SA), and oleic acid (OA) (250 μM) in the presence or absence of compound C (10 μM) for 24 h. (**A**,**B**) FGF21 concentrations in culture medium or cell lysates were measured by ELISA. Data are resented as the means ± SEM from 3 different sets of experiments. (**C,D**) Representative Western blots are shown on the left. Full-length blots are presented in Supplementary Figs S3 and S4. (**E**) Cultured neonatal rat cardiac myocytes were transfected with pGL3-mFgf21pro1-luc or TK-luc. Cells were then treated with either vehicle or ISO (10 μM), NE (10 μM), PA (250 μM) or SA (250 μM) before being harvested. Data are presented as mean ± SEM from 3 different sets of experiments on the right. Expression level of control is expressed as 1.0. *p < 0.05, **p < 0.01 compared with vehicle control (Con) or BSA, as measured by Tukey-Kramer test (**A,B**), Steel-Dwass test (**C,D**) or unpaired Student’s t-test (**E**).

As shown in Fig. 4, both isoproterenol (ISO) and norepinephrine (NE) significantly increased FGF21 levels in medium and in cell lysates (Fig. 4A). Saturated fatty acids (SFAs), PA and SA, but not unsaturated fatty acid, OA, induced FGF21 expression in medium and cell lysates (Fig. 4B). Consistent with the results of ELISA, Western blot analysis revealed that both ISO and NE induced FGF21 protein levels and phosphorylated form of AMPK and SIRT1 (Fig. 4C, Supplementary Fig. S3). Likewise, PA and SA but not OA up-regulated FGF21 protein levels and AMPK phosphorylation and SIRT1 expression (Fig. 4D, Supplementary Fig. S4).

Interestingly, ELISA revealed that an increase in FGF21 levels in the culture medium or in cell lysates from ISO- or NE-stimulated cardiac myocytes as well as from PA- or SA-stimulated cells was substantially attenuated by prior incubation with compound C (Fig. 4A,B). Western blot analysis showed that compound C substantially inhibits ISO- or NE-induced, and PA- or SA- induced FGF21 expression, AMPK phosphorylation, and ATF4 and SIRT1 expression (Fig. 4C,D, Supplementary Figs S3, S4). In addition, siRNA for AMPKα1 almost completely abrogated the inducible expression of FGF21, SIRT1 and PGC1α by AICAR, NE and PA. These results suggest that AMPK activation is required for catecholamine- and SFAs-induced FGF21, ATF4 and SIRT1 expression.

Then, the transient transfection assays of pGL3-mFgf21 pro1-luc reporter gene were performed. Results showed that luciferase activity of pGL3-mFgf21 pro1-luc is significantly induced by ISO, NE, PA, and SA, by ~3.1-, ~2.8-, ~1.9- and ~2.6-fold, respectively while no response was observed in TK-luc reporter gene (Fig. 4E). These results suggest that catecholamines and SFAs induce FGF21 expression at the transcriptional level.

### Induction of FGF21 and AMPK-dependent pathway by FGF21 stimulation

**Figure 5:**
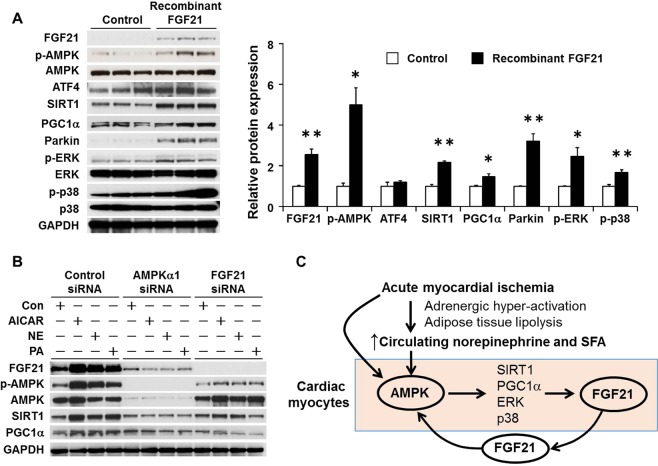
Activation of AMPK by recombinant FGF21 in the cardiac myocytes. (**A**) Representative Western blots in neonatal rat cardiac myocytes exposed to recombinant FGF21 (10 ng/mL) for 24 h are shown on the left. Full-length blots are presented in Supplementary Fig. S5. Data are presented as the means ± SEM from 2 different sets of experiments. Protein levels were normalized by GAPDH, and phosphorylation of proteins were normalized by total proteins in the same samples. Expression level of control is expressed as 1.0. *p < 0.05, **p < 0.01, as measured by Steel-Dwass test. (**B**) siRNA-mediated silencing of the expression of the AMPKα1 or FGF21 genes in neonatal rat cardiac myocytes and representative Western blots. Full-length blots are presented in Supplementary Fig. S6. (**C**) Proposed scheme for the feed-forward regulation of AMPK-FGF21 pathways in cardiac myocytes in response to acute ischemia. Upon onset of myocardial ischemia, circulating norepinephrine and SFAs concentrations rise following adrenergic hyper-activation and adipose tissue lipolysis. Elevated levels of norepinephrine and SFAs induce AMPK phosphorylation, which in turn activates down-stream targets of AMPK, such as SIRT1, PGC1α, ERK, p38 MAPK and FGF21. FGF21 then activates AMPK in the cardiac myocytes in an autocrine manner, and may contribute to mitochondrial homeostasis and cardioprotection.

Increasing number of evidence indicates that in addition to the role as energy sensor whose activation is to restore energy balance in myocardial ischemia^30^, AMPK plays a critical role in modulating several cellular processes that are critical for cell survival during ischemia. AMPK up-regulates the expression of antioxidants, stimulates autophagy, down-regulates proapoptotic proteins, and suppresses ER stress, all of which help to prevent myocardial cell death during prolonged ischemia^31,32^. Figure 5A and Supplementary Fig. S5 demonstrate that recombinant FGF21 (10 ng/mL) efficiently increased its own expression and AMPK phosphorylation. In addition, FGF21 activated multiple signaling molecules such as SIRT1, PGC1α, ERK, and p38 mitogen-activated protein kinase (MAPK). These results clearly indicate that cardiac myocytes are FGF21-responsive cells, as previously described^12,16^.

To directly test the role of AMPK and FGF21 in the activation of SIRT1 and PGC1α by AICAR, NE or PA, we employed siRNA-mediated knockdown of AMPK and FGF21 in the cultured cardiac myocytes. As shown in Fig. 5B, AMPKα1 siRNA, which mostly abolished the expression of AMPK, almost completely inhibited the induction of FGF21, SIRT1, and PGC1α expression by AICAR, NE and PA. These results strongly suggest that AMPKα1 is required for FGF21, SIRT1, and PGC1α expression in the cardiac myocytes. Likewise, siRNA for FGF21 significantly attenuated the induction of AMPK phosphorylation, SIRT1 and PGC1α. These results suggest that both AMPK and FGF21 are required for full activation of SIRT1 and PGC1α in response to AICAR, NE and PA.

Because SIRT1, PGC1α, p38 MAPK and Parkin serve as critical mediators of the cardioprotective effects of AMPK in the ischemic heart^33–35^, our results imply that FGF21 induces the AMPK-dependent cardioprotective mechanism in cardiac myocytes. Furthermore, together with the findings that FGF21 is induced by AMPK activation, cardiac AMPK-FGF21 pathway constitutes the feed-forward circuitry in response to ischemic stress (Fig. 5C).

## Discussion

Understanding the key signaling pathways that regulate cardiac energy metabolism and oxidative stress response may lead to the discovery of new therapeutic targets for patients with AMI. Here, we demonstrate from the clinical study that serum FGF21 is rapidly induced during early hours of AMI, and that peak of FGF21 levels is significantly correlated with that of FABP4, a protein secreted by adipose tissue lipolysis in response to norepinephrine stimulation^19^. In addition, we found that myocardial FGF21 production and release as well as activation of AMPK-FGF21 axis by heart are robustly induced upon myocardial ischemia in mice, and that activation of AMPK is required for catecholamine- and SFA-induced FGF21 expression in cardiac myocytes. Furthermore, FGF21 induces its own expression and the activation of AMPK-SIRT1-PGC-1α pathway, which has been implicated in cardioprotection in the ischemic heart^21–23^. These findings provide notion that catecholamine and its stimulation of adipose tissue lipolysis induce AMPK-FGF21-feed forward loop that potentially leads to the cardioprotection against ischemic damage.

Many studies have shown that circulating levels of FGF21 are elevated in patients with chronic cardiovascular diseases such as stable coronary artery disease^36^, carotid atherosclerosis^37^, hypertension^38^, persistent atrial fibrillation^39^ and diastolic heart failure^40^. However, few studies have described the effects of acute cardiovascular events on circulating FGF21 levels. Our present study demonstrates the prominent and rapid increase in circulating FGF21 in patients with AMI. This finding prompted us to speculate that ischemia-induced excessive sympathetic activation may evoke a signal to stimulate FGF21 expression. Three independent lines of observations support this hypothesis. First, in the AMI group, peak FGF21 levels were significantly correlated with that of FABP4 (r = 0.425, p = 0.002). In our previous studies, FABP4, which has originally been identified as intracellular lipid chaperone that mediates lipid metabolism and inflammation^41^, was rapidly and robustly increased in the patients with AMI in general, and in patients complicated with out of hospital cardiac arrest (OHCA) irrespective of the severity of myocardial ischemia in particular^19^. Indeed, our *in vitro* data showed that FABP4 is released from adipocytes by adrenergic β3 stimulation^19^. Second, we previously reported that serum FABP4 levels were strongly correlated with serum norepinephrine levels during exercise in humans^18^. Third, our present *in vitro* study revealed that catecholamines (ISO, NE) and SFAs (PA, SA) stimulate the expression and secretion of FGF21 in the cultured cardiac myocytes. These data lend support to our hypothesis that rapid increase in serum FGF21 levels in AMI could be, at least in part, attributed to adrenergic hyperactivity and resultant lipolysis. Intriguingly, these findings will provide insight on whether FGF21 could be used as a biomarker for integrated metabolic stress provoked by AMI.

Noteworthy, our data indicate that catecholamines and SFAs activate AMPK-FGF21 pathway in cardiac myocytes. Catecholamines induce serious arrhythmias and myocardial damage, both directly and indirectly, as a consequence of catecholamine-induced elevation of circulating FFA in AMI^42,43^, whereas few studies have described the potentials of catecholamines to directly induce cardioprotective mechanisms in the ischemic heart. Likewise, to our best of our knowledge, few studies reported an activation of cardioprotective pathways by SFAs, which are well known to induce lipotoxicity when excessive SFAs are utilized for mitochondrial FA oxidation^44^. Although precise molecular mechanisms underlying the activation of AMPK-FGF21 pathway by catecholamines and SFAs remain to be determined, it is likely that mitochondrial ROS (mROS) can activate this pathway, via mechanisms that involve alterations in energy production and the AMP/ATP ratio. This hypothesis is supported by recent studies indicating that AMPK plays a crucial role in maintaining the cellular metabolic balance in response to increased mROS production^45–47^. In addition, it has been well recognized that both catecholamines and SFAs increase mROS production in the heart^48–50^. The role of mROS in catecholamine- and SFA-mediated AMPK activation in ischemic heart will be explored in future work.

What is the pathophysiological relevance of AMPK-FGF21 activation in early stage of AMI? There may be some concern that activation of AMPK might be detrimental for ischemic heart because activation of AMPK could potentially accelerate glycolysis and fatty acid oxidation at the expense of glucose oxidation which consequently lead to increased lactate production and intracellular acidosis^51^. However, the activation of AMPK-FGF21 pathway is likely to be adaptive to ischemic myocardium considering a favorable action of AMPK in the energy-stressed heart^32,47,52^.

Previous studies identified FGF21-induced multiple intracellular signaling pathways in the heart, including phosphatidylinositide 3-kinase (PI3K)-Akt-signaling pathway in ischemic heart^53^, ERK1/2 and p38 MAPK pathway in diabetic heart^54^, and ERK1/2 and CREB-PGC1α pathway in hypertrophied heart^16^. Our findings that that FGF21 activates AMPK phosphorylation, and induces expression of type III NAD+ -dependent deacetylase SIRT1 and transcriptional coactivator PGC1α are consistent with the results reported in adipocytes and skeletal muscle cells, in which FGF21 induces AMPK-SIRT1-PGC1α pathways to regulate mitochondrial and fatty acid oxidation in response to energy demand^33,34,55,56^. FGF21-induced AMPK-SIRT1-PGC1α pathway also appears to play an important cardioprotective role for ischemia/reperfusion injury because SIRT1 deacetylation of PGC1α activation leads to an induction of anti-oxidant genes such as manganese superoxide dismutase (MnSOD) and thioredoxin-1 (Trx1), and a suppression of proapoptotic molecule such as Bax and cleaved caspase-3^23^. In addition, we observed that Parkin, which is cytosolic E3 ubiquitin ligase critical for mitochondrial quality control^25,26^, is induced in ischemic heart *in vivo* and in cardiac myocytes stimulated with FGF21 *in vitro*. Furthermore, we found that p38 MAPK is activated by FGF21 *in vitro*. Previous study demonstrated that p38 MAPK is a downstream effector of AMPK, and AMPK-induced glucose transport during ischemia is partially mediated by p38 MAPK pathway^35^. Although the results of our present study do not address whether AMPK-FGF21 axis functions as cardioprotective pathway, an induction of the members of AMPK targets by FGF21 expand on the concept that activation of AMPK is adaptive in response to the disruption of energy homeostasis and mitochondrial function in ischemic heart. With respect to energy production, FGF21 may serve as an inducer of fatty acid oxidation rather than glucose oxidation, because FGF21 knockdown impairs FAO and conversely increases glucose uptake during adaptive hypertrophy in mice^57^. To determine the precise role of FGF21 on utilization and preference of energy substrate in ischemic myocardium, further studies are required.

In addition, in collaboration with the finding that AMP-analog AMPK activator AICAR strongly induced FGF21, our data imply that AMPK-FGF21 pathway constitutes the feed-forward circuitry, which serves as potent and long-lasting protective signaling pathways against ischemic stress.

## Conclusions

The present study demonstrates that serum FGF21 levels are rapidly elevated upon onset of AMI, in parallel with adipose tissue lipolysis byproducts FABP4 and SFAs. *In vitro* study revealed that cardiac production and release of FGF21 are induced by catecholamine and SFAs via AMPK activation, and FGF21 in turn activates FGF21 expression and AMPK/SIRT1/PGC1α pathway. The integral role that AMPK plays in energy homeostasis and cell survival in ischemic myocardium suggest that AMPK-FGF21 feed-forward loop may be a potential therapeutic target to limit myocardial cell damage.

## Methods

### Study patients

We studied 50 patients with a final diagnosis of AMI according to the redefined European Society of Cardiology/American College of Cardiology committee criteria^58^, who underwent successful reperfusion by primary PCI. In addition, 43 patients with stable AP who were scheduled to undergo PCI for 75% or greater organic stenosis of at least one major coronary artery were also included in this study. Patients with either AMI or stable AP were eligible for this study if their characteristics were assessed by a detailed medical history and physical examination during hospitalization. We excluded patients with end-stage renal disease receiving hemodialysis and those who were admitted more than 24 hours after the onset of chest pain.

All participants signed informed consent forms when their clinical conditions allowed them to provide free and unbiased consent. This study was carried out in accordance with the principles of the Declaration of Helsinki, and the study protocol was approved by the Institutional Review Board of Gunma University Hospital.

### Measurement of biochemical variables

Blood samples were collected on admission to the hospital for patients with AMI or at the time of scheduled admission for the stable AP patients who undergo elective PCI. After samples were immediately processed to separate serum and were used for quantitative determination of conventional laboratory metabolites (LDL-cholesterol, HDL-cholesterol, triglyceride, glucose), serum was stored at −30 °C until further analysis. Concentrations of FGF21 and FABP4 were quantified by using commercially available enzyme-linked immunosorbent assay (ELISA) kits for human FGF21 (R&D Systems) and human FABP4 (Biovendor), respectively. As specified by the manufacturer, the lower limit of detection of serum FGF21 was 4.67 pg/mL and FABP4 was 0.05 ng/mL. cTnT concentrations were determined with the use of high-sensitivity electrochemiluminescence assays in the Elecysy-2010 system (Roche Diagnostics). FFA concentrations were determined by enzyme-linked assays and individual fatty acids were analyzed by capillary gas chromatography at SRL Inc., and were expressed as a percentage of total fatty acids. We evaluated the time course of the changes in the circulating FGF21, FABP4, FFA, and cTnT levels in a subgroup of AMI patients whose blood samples could be serially collected on admission, immediately after emergent PCI, at 3, 6 h and 1 day after PCI.

### *In vivo* studies of ligation of the coronary artery

C57BL/6 strain (WT) mice were purchased from CLEA Japan Inc. Male 8- to10-week-old mice, weighing 23–25 g, were subjected to left anterior descending coronary artery ligation to model an MI, as described^59^, with minor modifications^60^. Briefly, mice were anesthetized with isoflurane, intubated, and ventilated with a volume-controlled ventilator (KN-58 SLA ventilator; Natsume Factory Inc.). A left thoracotomy was performed with an incision between the third and fourth intercostal spaces and the left anterior descending branch of the coronary artery was visualized. the pericardium was opened, and the proximal left anterior descending coronary artery was ligated with an 8–0 nylon suture (Natsume Factory Inc.). The chest wall was then closed in layers using 5-0 nylon sutures. Mice were sacrificed 1 h and 24 h after this injury, and the hearts were isolated. Sham-operated control animals underwent a similar surgery without ligation of the artery. Serum levels of FGF21 were measured using an mouse ELISA kit, according to the manufacturer’s protocol (R&D Systems).

Animal experiments using these mice were approved by and performed according to the guidelines of the Committee of Experimental Animal Research of Gunma University.

### Preparation of neonatal rat cardiac myocytes

Primary neonatal rat cardiac myocyte cultures were prepared as previously described^61^. Using this method, we routinely obtained cardiac myocyte-rich cultures with >95% of the cells being cardiac myocytes, as assessed by immunocytochemical staining with monoclonal antibody against sarcomeric α-actinin (Sigma-Aldrich).

Serum-starved (24 h) cardiac myocytes with 0.1% BSA (24 h) were incubated in the presence or absence of pan-β-adrenergic receptor agonist isoproterenol (ISO; 10 μM), norepinephrine (NE; 10 μM), fatty acid-free BSA (Sigma-Aldrich)-conjugated palmitic acid (PA), stearic acid (SA) and oleic acid (OA) (Sigma, 250 μM). In experiments determining the effects of AMPK on FGF21 expression, serum-starved cardiac myocytes were stimulated with 1 mM 5-aminoimidazole-4-carboxamide ribonucleotide (AICAR, Toronto Research Chemicals), a cell-permeable adenosine analogue^62^, known AMPK activator for 24 h.

The effect of inhibiting AMPK on AICAR-stimulated FGF21 expression was determined by preincubating neonatal rat cardiac myocytes for 24 h with 10 μM of 6-[4-(2-piperidin-1-yl-ethoxy)-phenyl]-3-pyridin-4-yl-pyrrazolo-[1,5-a]pyrimidine (compound C, (Merck), a competitive inhibitor of AMPK^27^.

AMPKα1 siRNA and FGF21 siRNA were purchased from Santa Cruz Biotechnology (AMPKα1: sc-270142; FGF21: sc-156171) and transfected with Lipofectamine RNAiMAX Regent (Invitrogen) according to the manufacturer’s protocol. siGFP concentration used was 50 nM, which has little, if any, off-target effects. After 24 h transfection, the cells were serum starved for another 24 h.

### Luciferase assays

Cultured neonatal rat cardiac myocytes were transfected with the luciferase reporter plasmid pGL3-mFgf21pro1-luc constructed by S. Oyadomari^63^ (addgene plasmid 101797) or control thymidine kinase (TK). The cells were incubated 48 h before being harvested with Passive Lysis buffer (Promega). Luciferase activity was measured with luciferase assay substrate (Promega) using luciferase reporter assay system (Promega) as described previously^64^.

### Western blot analysis

Tissue samples and cells were homogenized on ice in RIPA buffer (20 mM Tris-HCl [pH 7.4], 150 mM NaCl, 1% NP-40, 1% sodium deoxycholate, 0.1% SDS, and containing complete mini and phosSTOP solution (Roche)). The mixture was centrifuged at 15,000 rpm for 30 min and the supernatant was subjected to SDS-PAGE. Protein concentrations were determined by the Bradford method using a colorimetric assay (Bio-Rad).

Western blot analysis was performed according to standard procedures using the following primary antibodies: rabbit monoclonal FGF21 (Abcam, ab171941, 1:250), phospho- AMPKα (Thr172; Cell Signaling Technology, #2535, 1:250), AMPK (Cell Signaling Technology, #2603, 1:500), ATF4 (Cell Signaling Technology, #11815, 1:250), SIRT1 (Cell Signaling Technology, #9475, 1:250), and GAPDH (Cell Signaling Technology, #2118, 1:500). Antigens were revealed by Immobilon Western HRP Substrate (Millipore) after an incubation with horseradish peroxidase-conjugated anti-rabbit or mouse IgG. The density of a band was quantified using ImageJ software.

### Statistical analysis

All continuous variables are presented as the mean ± standard error of the mean (SEM), unless otherwise specified. A two-group comparison was performed using the Mann-Whitney U-test or unpaired Student’s t-test, while a multiple-group comparison was performed by a 1-way ANOVA with Steel-Dwass or Tukey-Kramer’s multiple comparison tests. Time profiles for serum FGF21, FABP4, cTnT and FFA were analysed using Friedman’s tests. Then, univariate and multivariable linear regression analyses were used to determine the contributions of these variables to peak FGF21 levels. A two-sided p-value < 0.05 was considered statistically significant. *p < 0.05, **p < 0.01, ***p < 0.001.

## Supplementary information

Supplemental data
