# Supplementary material for: Activation of cardiac AMPK-FGF21 feed-forward loop in acute myocardial infarction: Role of adrenergic overdrive and lipolysis byproducts
Source: Sci Rep. 2019 Aug 14;9:11841. doi: 10.1038/s41598-019-48356-1 (PMC6694166; doi:10.1038/s41598-019-48356-1)
Supplement: Supplementary file 1 — Supplemental data [file 41598_2019_48356_MOESM1_ESM.pdf]

Activation of cardiac AMPK-FGF21 feed-forward loop in acute myocardial infarction: Role of adrenergic overdrive and lipolysis byproducts

Hiroaki Sunaga[1], Norimichi Koitabashi[1], Tatsuya Iso[1],

Hiroki Matsui[2], Masaru Obokata[1], Ryo Kawakami[1], Masami Murakami[3],

Tomoyuki Yokoyama[2], and *Masahiko Kurabayashi[1]

[1]Department of Cardiovascular Medicine, Gunma University Graduate School of Medicine, Maebashi, Gunma, Japan; [2]Department of Laboratory Sciences, Gunma University Graduate School of Health Sciences, Maebashi, Gunma, Japan; [3] Department of Clinical Laboratory Medicine, Gunma University Graduate School of Medicine, Maebashi, Gunma, Japan

Address for Correspondence

*Masahiko Kurabayashi, MD, PhD

Department of Cardiovascular Medicine, Gunma University Graduate School of Medicine,

3-39-15 Showa-machi, Maebashi, Gunma 371-8511, Japan

Tel.: +81-27-220-8140; Fax: +81-27-220-8150; E-mail address: mkuraba@gunma-u.ac.jp

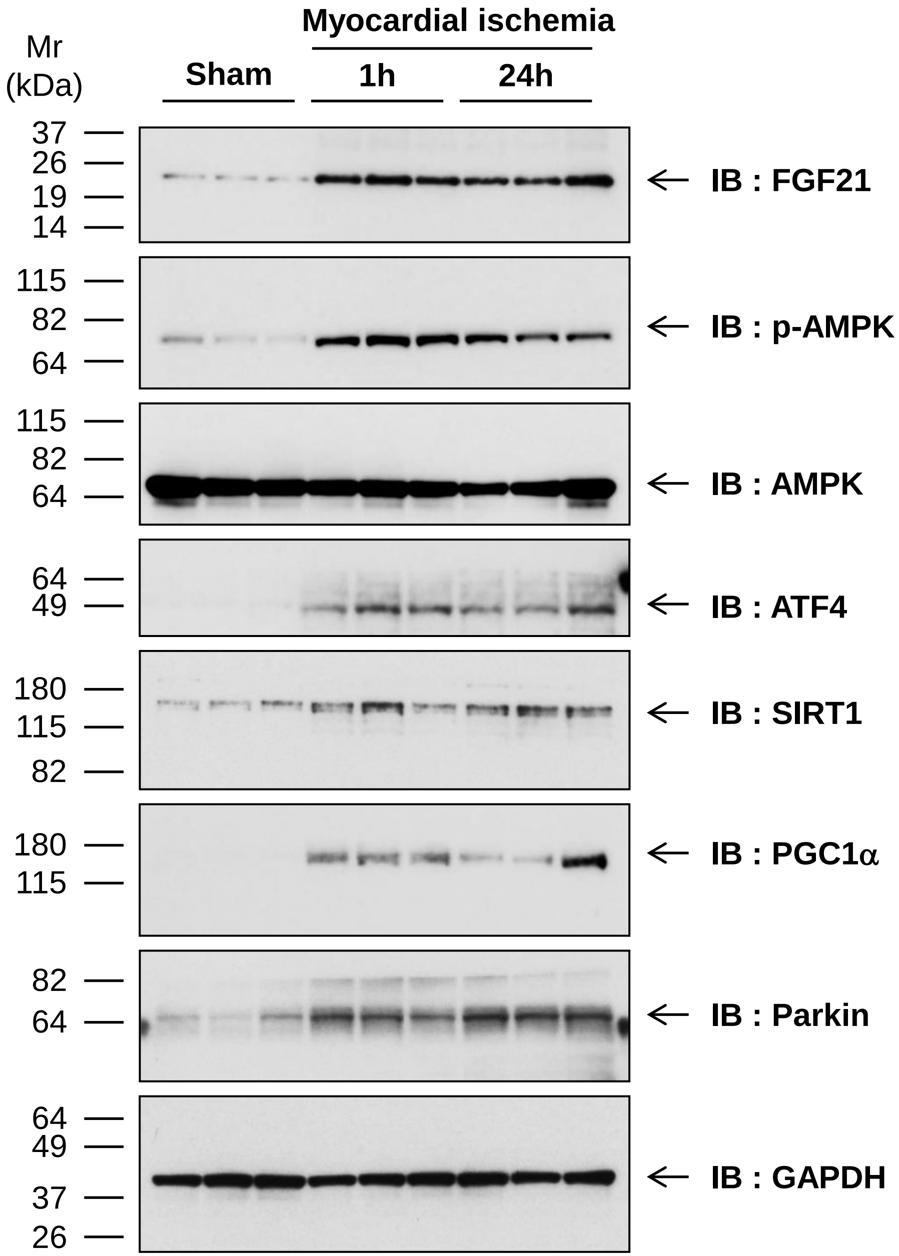
Mr (kDa)
Myocardial ischemia
Sham
1h
24h
37
26
19
14
IB : FGF21
115
82
64
IB : p-AMPK
115
82
64
IB : AMPK
64
49
IB : ATF4
180
115
82
IB : SIRT1
180
115
IB : PGC1α
82
64
IB : Parkin
64
49
37
26
IB : GAPDH

Supplementary Figure Legends

Supplementary Figure S1. Full-length Western blot of Figure 2B .

Full-length representative Western blots are shown. The blots were exposed on the autoradiography film then developed with Fuji Medical Film Processor FPM100, changed to appropriate grey background using Microsoft PowerPoint. This images were inserted into Fig. 2B of the main article.

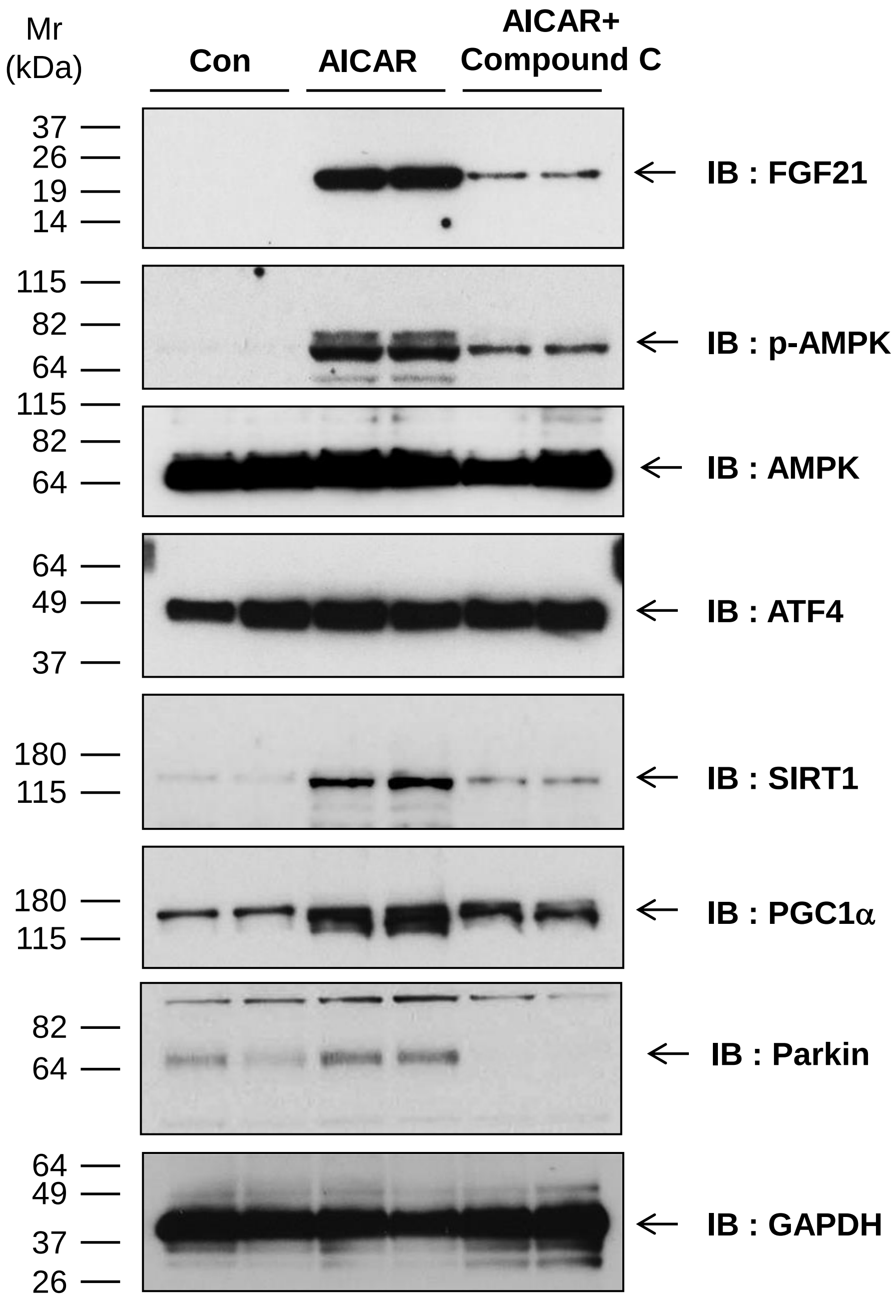
Mr (kDa)
Con
AICAR
AICAR+ Compound C
37
26
19
14
IB : FGF21
115
82
64
IB : p-AMPK
115
82
64
IB : AMPK
64
49
37
IB : ATF4
180
115
IB : SIRT1
180
115
IB : PGC1α
82
64
IB : Parkin
64
49
37
26
IB : GAPDH

Supplementary Figure S2. Full-length Western blot of Figure 3B .

Full-length representative Western blots are shown. The blots were exposed on the autoradiography film then developed with Fuji Medical Film Processor FPM100, changed to appropriate grey background using Microsoft PowerPoint. This images were inserted into Fig. 3B of the main article.

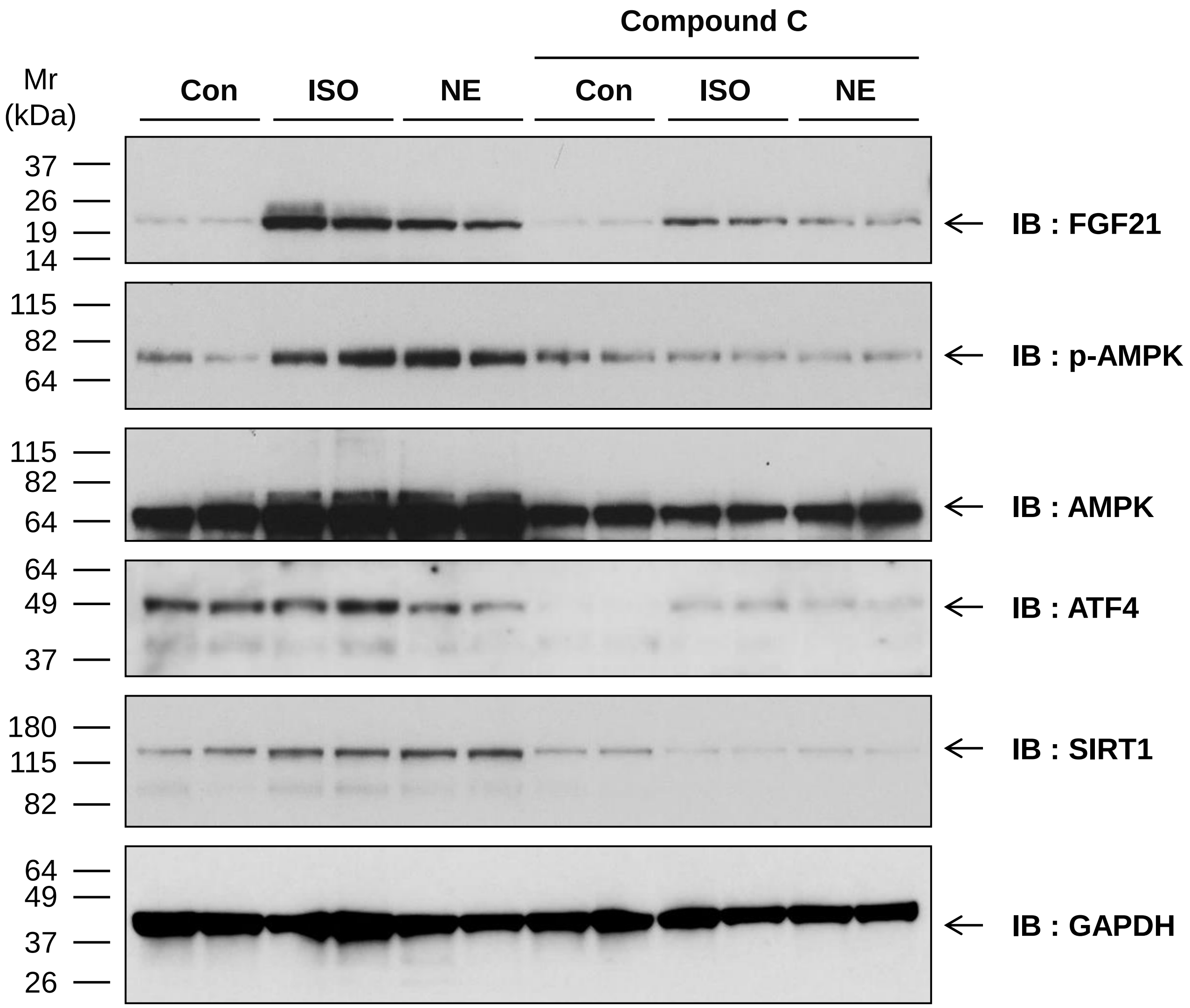
Compound C
Mr
(kDa)
Con
ISO
NE
Con
ISO
NE
37
26
19
14
IB : FGF21
115
82
64
IB : p-AMPK
115
82
64
IB : AMPK
64
49
37
IB : ATF4
180
115
82
IB : SIRT1
64
49
37
26
IB : GAPDH

Supplementary Figure S3. Full-length Western blot of Figure 4C .

Full-length representative Western blots are shown. The blots were exposed on the autoradiography film then developed with Fuji Medical Film Processor FPM100, changed to appropriate grey background using Microsoft PowerPoint. This images were inserted into Fig. 4C of the main article.

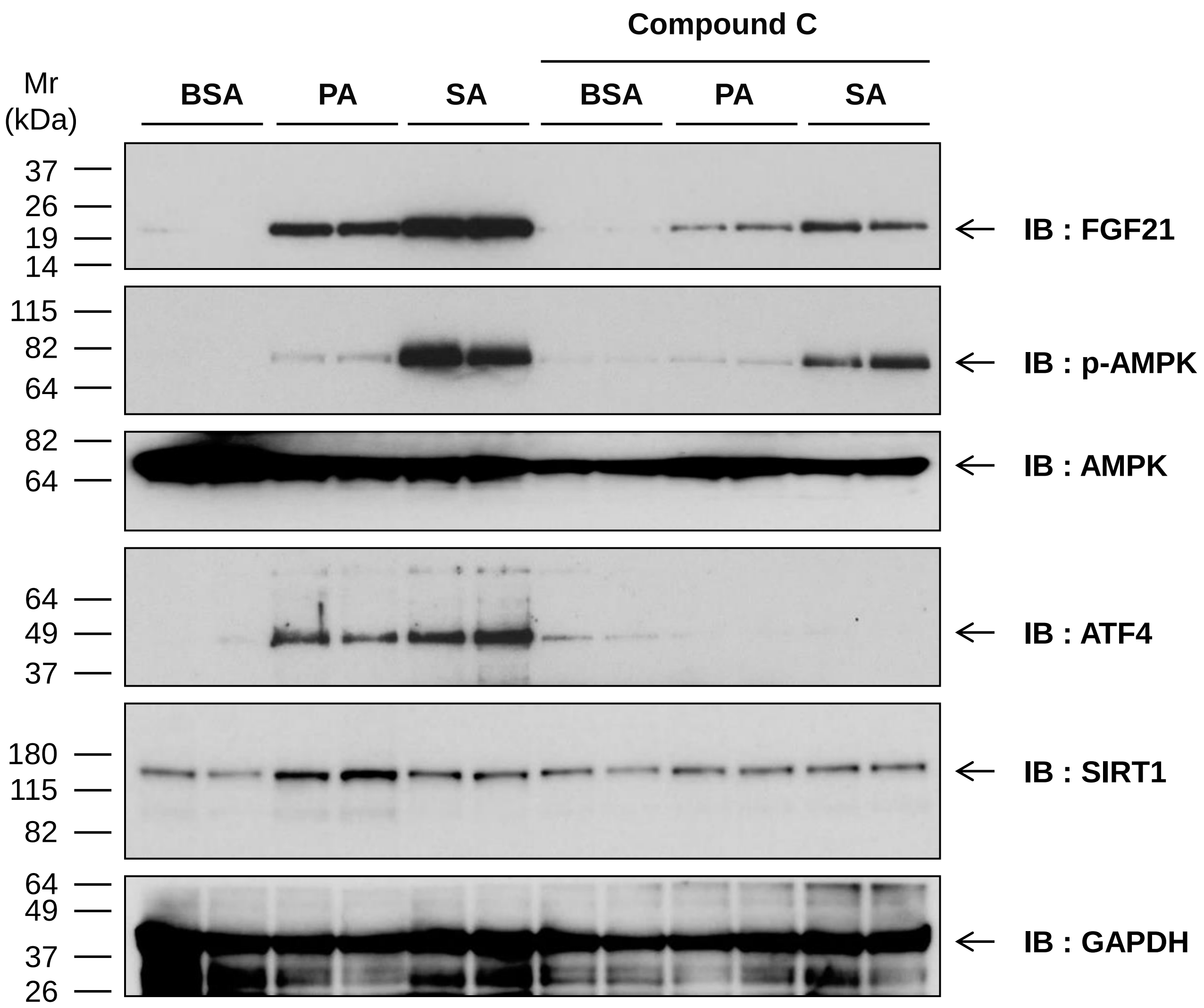
Compound C
Mr (kDa)
BSA
PA
SA
BSA
PA
SA
37
26
19
14
115
82
64
82
64
64
49
37
180
115
82
64
49
37
26
IB : FGF21
IB : p-AMPK
IB : AMPK
IB : ATF4
IB : SIRT1
IB : GAPDH

Supplementary Figure S4. Full-length Western blot of Figure 4D .

Full-length representative Western blots are shown. The blots were exposed on the autoradiography film then developed with Fuji Medical Film Processor FPM100, changed to appropriate grey background using Microsoft PowerPoint. This images were inserted into Fig. 4D of the main article.

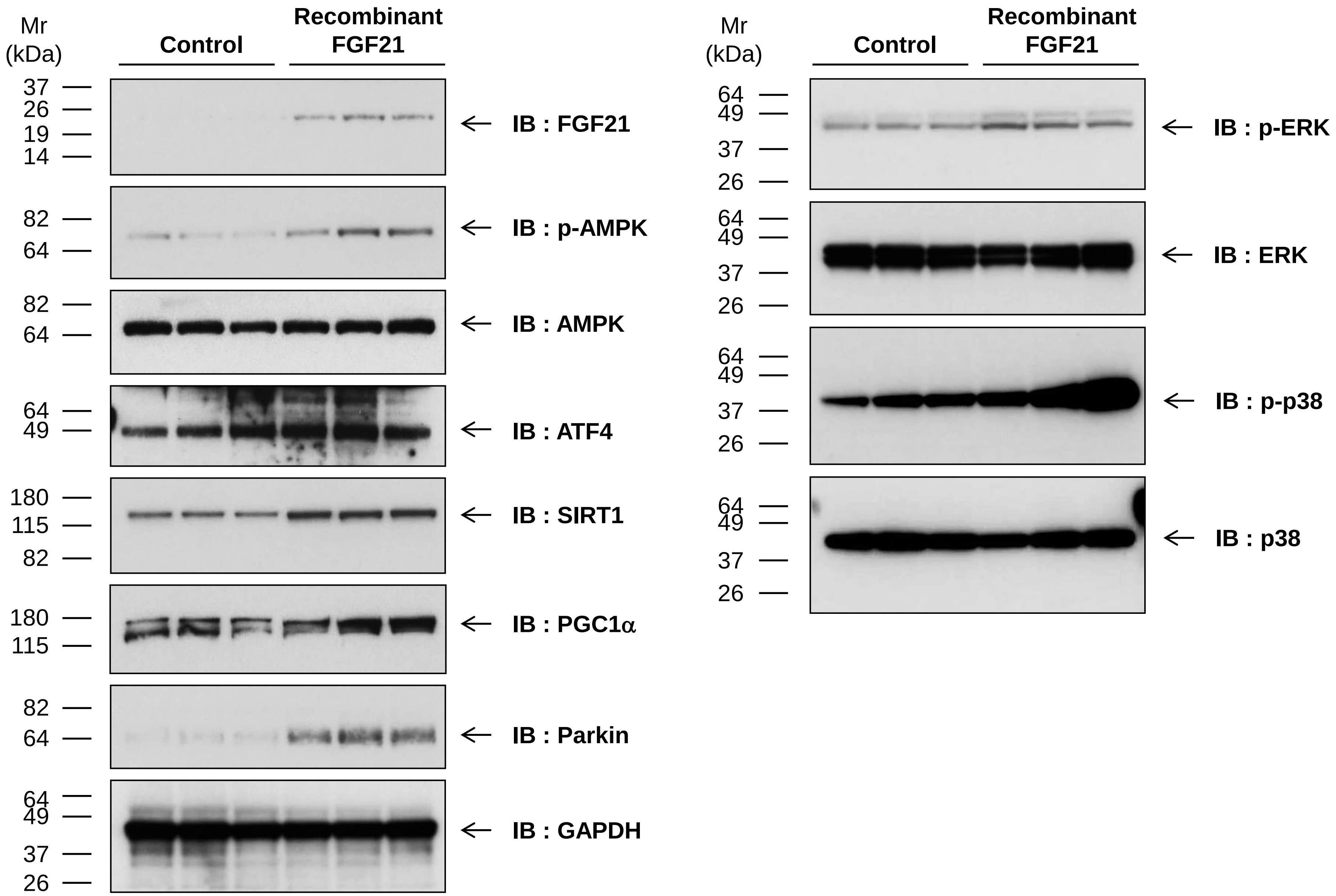
Mr (kDa)
Control
Recombinant FGF21
37
26
19
14
IB : FGF21
82
64
IB : p-AMPK
82
64
IB : AMPK
64
49
IB : ATF4
180
115
82
IB : SIRT1
180
115
IB : PGC1α
82
64
IB : Parkin
64
49
37
26
IB : GAPDH
Mr (kDa)
Control
Recombinant FGF21
64
49
37
26
IB : p-ERK
64
49
37
26
IB : ERK
64
49
37
26
IB : p-p38
64
49
37
26
IB : p38

Supplementary Figure S5. Full-length Western blot of Figure 5A .

Full-length representative Western blots are shown. The blots were exposed on the autoradiography film then developed with Fuji Medical Film Processor FPM100, changed to appropriate grey background using Microsoft PowerPoint. This images were inserted into Fig. 5A of the main article.

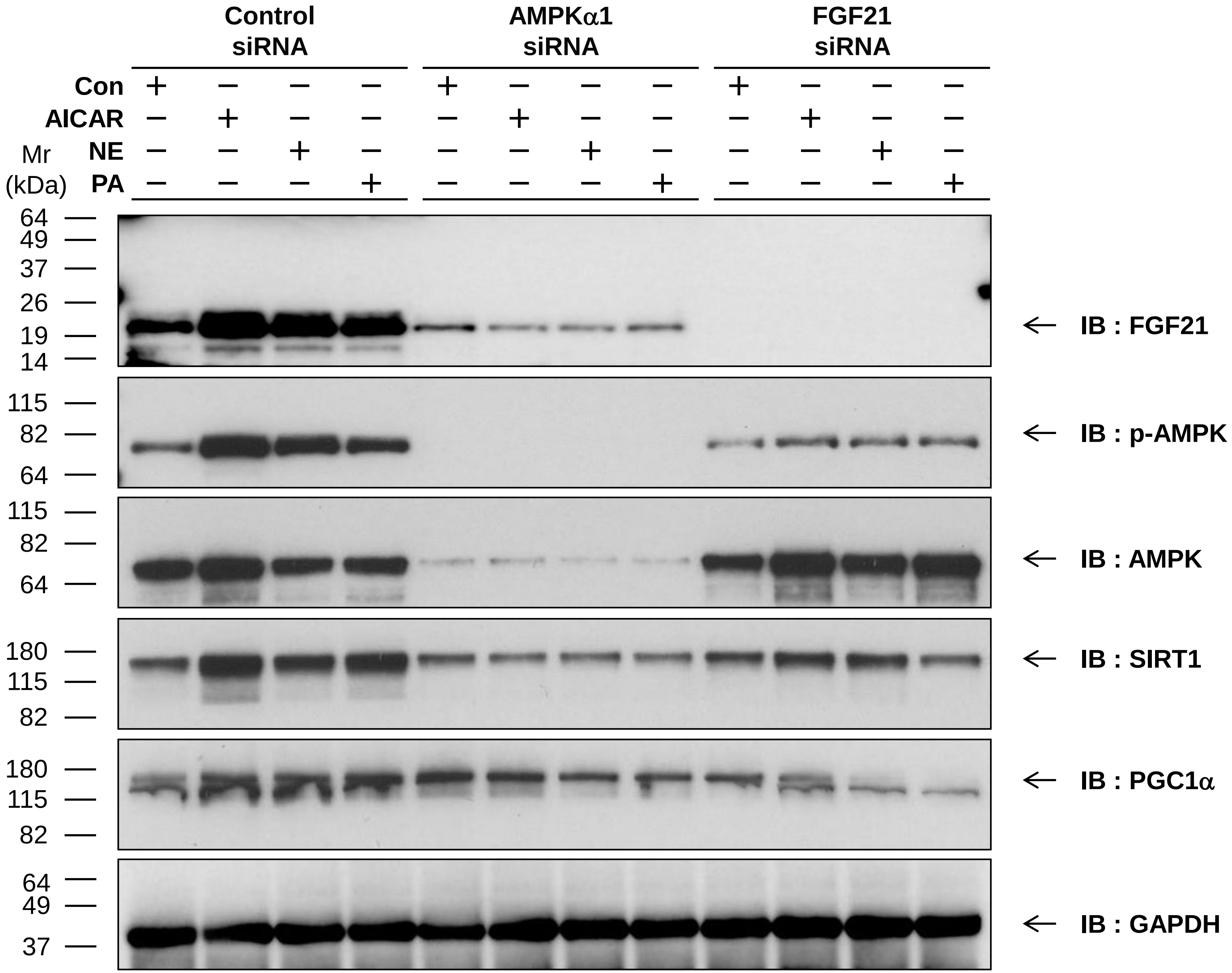

Control siRNA
AMPKα1 siRNA
FGF21 siRNA
Con
AICAR
NE
PA
Mr (kDa)
64
49
37
26
19
14
115
82
64
115
82
64
180
115
82
180
115
82
64
49
37
IB : FGF21
IB : p-AMPK
IB : AMPK
IB : SIRT1
IB : PGC1α
IB : GAPDH

Supplementary Figure S6. Full-length Western blot of Figure 5B .

Full-length representative Western blots are shown. The blots were exposed on the autoradiography film then developed with Fuji Medical Film Processor FPM100, changed to appropriate grey background using Microsoft PowerPoint. This images were inserted into Fig. 5B of the main article.

Supplementary Table S1. Comparison of serum fatty acid profile (as percentage of total fatty acids) between sAP and AMI group.

	stable AP (n=30)	AMI (n=34)	p
C12:0	0.186 ± 0.234	0.270 ± 0.616	0.475
C14:0	0.944 ± 0.458	0.866 ± 0.365	0.466
C14:1n-5	0.067 ± 0.041	0.052 ± 0.031	0.100
C16:0 (palmitic acid)	24.406 ± 1.944	27.305 ± 1.629	<0.001
C16:1n-7	2.562 ± 0.991	2.971 ± 2.291	0.357
C18:0 (stearic acid)	7.637 ± 1.168	8.735 ± 1.412	<0.001
C18:1n-9 (oleic acid)	26.576 ± 3.112	23.220 ± 4.367	<0.001
C18:2n-6 (linoleic acid)	24.280 ± 3.630	23.063 ± 3.686	0.196
C18:3n-6 (γ-linolenic acid)	0.265 ± 0.145	0.178 ± 0.087	0.007
C18:3n-3	0.656 ± 0.254	0.547 ± 0.193	0.065
C20:0	0.297 ± 0.052	0.349 ± 0.142	0.056
C20:1n-9	0.226 ± 0.065	0.228 ± 0.060	0.918
C20:2n-6	0.245 ± 0.033	0.256 ± 0.030	0.178
C20:3n-9	0.102 ± 0.054	0.088 ± 0.031	0.216
C20:3n-6	1.098 ± 0.332	0.970 ± 0.311	0.120
C20:4n-6 (AA, arachidonic acid)	3.383 ± 0.654	3.959 ± 1.242	0.024
C20:5n-3 (EPA, eicosapentaenoic acid)	1.584 ± 0.839	1.085 ± 0.513	0.008
C22:0	0.649 ± 0.137	0.795 ± 0.465	0.094
C22:1n-9	0.038 ± 0.029	0.052 ± 0.041	0.119
C22:4n-6	0.103 ± 0.034	0.07 ± 0.042	0.001
C22:5n-3	0.363 ± 0.115	0.321 ± 0.148	0.2195
C24:0	0.634 ± 0.154	0.762 + 0.429	0.1165
C22:6n-3 (DHA, docosahexaenoic acid)	2.384 ± 0.719	2.022 ± 0.647	0.0428
C24:1n-9	1.484 ± 0.502	1.850 ± 0.772	0.0292
EPA/AA	0.496 ± 0.314	0.279 ± 0.105	0.0011
DHA/AA	0.744 ± 0.315	0.538 ± 0.169	0.0030
(EPA + DHA) /AA	1.240 ± 0.577	0.817 ± 0.249	0.0008
